# Management of the Insane in Texas

**Published:** 1888-04

**Authors:** 


### THE MANAGEMENT OF THE INSANE IN TEXAS.

### DR. DENTON'S PAPER.

For plain language, void of ambiquity or circumlocution, this paper excells. While we commend it to our readers for its soundness and practical value, we blush to know that the outside world will see in it only a sad commentary on the intelligence of the people of this great State.

Dr. Denton shows that during the thirty years, since the foundation of the State Lunatic Asylum, fifteen changes of Superintendent have been made, and, with one or two exceptions, by removal by the Governor; and says "all these changes have been made without the assignment of a single cause, or the preferment of a single charge, so far as the record shows." By way of contrast, and as showing the consistency (?) of our blessed Governors—that rare jewel—he might have cited the fact that in the *only* instance in which charges of a grave nature had been preferred against a Superintendent, and a verdict of guilty (in part) returned by the Board; —the only instance where, in the opinion of a majority of the Board, "good and sufficient cause" for removal was thought to exist, *no* removal followed; but changes in the composition of the Board followed, as plentifully as "autumn leaves in the fall."

It will be seen, by reading the proceedings of the Austin District Medical Society, that as an outcome of Dr. Denton's paper, a resolution was adopted, asking the co-operation of the State Medical Association in an effort to procure the passage of a law to remedy the great evil complained of in this paper, and which is patent. Doubtless action will be had on the resolution, at Galveston, when the JOURNAL will have something to say on the subject.

